# TRYPTOPHAN: A POTENTIAL BIOMARKER DISTINGUISHING CHRONIC VENOUS LEG ULCERS HEALING IN OLDER ADULTS

**DOI:** 10.1093/geroni/igad104.3674

**Published:** 2023-12-21

**Authors:** Jung Lyun Kim, Joyce Stechmiller, Michael Weaver, Debra Lyon, Timothy Garrett, Debra Kelly

**Affiliations:** Chungnam National University, Daejeon, Republic of Korea; University of Florida, Gainesville, Florida, United States; University of Florida, Gainesville, Florida, United States; University of Florida, Gainesville, Florida, United States; University of Florida, Gainesville, Florida, United States; University of Florida, Gainesville, Florida, United States

## Abstract

Chronic venous leg ulcers (CVLUs) affect 2 million persons annually, including 4% of people over age 65 years. Chronic wound healing is a complex process that is still not well understood. Indoleamine 2,3-dioxygenase (IDO) activity is highly active in chronic wounds. The study aims to examine the tryptophan (TRP) L-Kynurenine (KYN) metabolic pathway as a biomarker of CVLUs healing. We collected 60 serum samples from 30 older adult patients with CVLUs receiving weekly sharp debridement at a university wound clinic. Serum samples were collected at baseline, week 4, and week 8 until wound closure. Liquid chromatography-mass spectrometry (LC-MS) metabolomics was utilized to examine targeted metabolites. A robust Bayesian approach was employed to examine correlations between change of metabolites and linear healing slope. The mean age was 71.13 (±9.46); 25 (83.3%) were white; the mean wound area was 2823.54 (±6002.93) mm2; the mean wound duration was 341.67 (±538.29) days. A total of 11 participants had healed wounds during the study period. Those with healing CVLUs had higher levels of mean TRP at baseline and over time compared to individuals with non-healing CVLUs. Change in Kynurenic Acid (r:-0.36, Bayes factor: 3.70, 95% Credibility Interval [-0.62, -0.06]) was associated with a linear healing slope. Serum tryptophan may serve as a candidate biomarker for predicting wound healing trajectories, which can guide clinicians in making treatment decisions. Specifically, decreasing metabolites associated with the downstream activity of the kynurenine pathway, a pathway for tryptophan metabolism may be an indicator of healing under sharp debridement in CVLUs.

